# Dysregulation of the Wnt/β-catenin signaling pathway via Rnf146 upregulation in a VPA-induced mouse model of autism spectrum disorder

**DOI:** 10.1038/s12276-023-01065-2

**Published:** 2023-08-01

**Authors:** Gaeun Park, Wooyoung Eric Jang, Seoyeon Kim, Edson Luck Gonzales, Jungeun Ji, Seunghwan Choi, Yujin Kim, Ji Hwan Park, Hazara Begum Mohammad, Geul Bang, Minkyung Kang, Soobin Kim, Se Jin Jeon, Jin Young Kim, Kwang Pyo Kim, Chan Young Shin, Joon-Yong An, Min-Sik Kim, Yong-Seok Lee

**Affiliations:** 1 Department of Biomedical Science, Seoul National University College of Medicine, Seoul, 03080 Republic of Korea; 2 Department of Physiology, Seoul National University College of Medicine, Seoul, 03080 Republic of Korea; 3 Department of Applied Chemistry, Institute of Natural Science, Global Center for Pharmaceutical Ingredient Materials, Kyung Hee University, Yongin, 17104 Republic of Korea; 4 Department of Integrated Biomedical and Life Science, Korea University, Seoul, 02841 Republic of Korea; 5 BK21FOUR R&E Center for Learning Health Systems, Korea University, Seoul, 02841 Republic of Korea; 6 School of Medicine and Center for Neuroscience Research, Konkuk University, Seoul, 05029 Republic of Korea; 7 School of Biosystem and Biomedical Science, College of Health Science, Korea University, Seoul, 02841 Republic of Korea; 8 Department of New Biology, DGIST, Daegu, 42988 Republic of Korea; 9 Research Center for Bioconvergence Analysis, Korea Basic Science Institute, Ochang, 28119 Republic of Korea; 10 Department of Biomedical Science and Technology, Kyung Hee Medical Science Research Institute, Kyung Hee University, Seoul, 02447 Republic of Korea; 11 New Biology Research Center, DGIST, Daegu, 42988 Republic of Korea; 12 Center for Cell Fate Reprogramming and Control, DGIST, Daegu, 42988 Republic of Korea; 13 Neuroscience Research Institute, Seoul National University College of Medicine, Seoul, 03080 Republic of Korea; 14 Wide River Institute of Immunology, Seoul National University, Hongcheon, 25159 Republic of Korea

**Keywords:** Autism spectrum disorders, Proteomics

## Abstract

Autism spectrum disorder (ASD) is a neurodevelopmental disorder associated with impaired social behavior and communication, repetitive behaviors, and restricted interests. In addition to genetic factors, environmental factors such as prenatal drug exposure contribute to the development of ASD. However, how those prenatal factors induce behavioral deficits in the adult stage is not clear. To elucidate ASD pathogenesis at the molecular level, we performed a high-resolution mass spectrometry-based quantitative proteomic analysis on the prefrontal cortex (PFC) of mice exposed to valproic acid (VPA) in utero, a widely used animal model of ASD. Differentially expressed proteins (DEPs) in VPA-exposed mice showed significant overlap with ASD risk genes, including differentially expressed genes from the postmortem cortex of ASD patients. Functional annotations of the DEPs revealed significant enrichment in the Wnt/β-catenin signaling pathway, which is dysregulated by the upregulation of Rnf146 in VPA-exposed mice. Consistently, overexpressing Rnf146 in the PFC impaired social behaviors and altered the Wnt signaling pathway in adult mice. Furthermore, Rnf146-overexpressing PFC neurons showed increased excitatory synaptic transmission, which may underlie impaired social behavior. These results demonstrate that Rnf146 is critical for social behavior and that dysregulation of Rnf146 underlies social deficits in VPA-exposed mice.

## Autism spectrum disorder: Improving understanding at the molecular level

Increased levels of a protein that regulates neuronal signaling pathway, called Rnf146, are associated with social behavior deficits consistent with autism spectrum disorder (ASD) in a mouse model. Individuals with ASD show decreased sociability, repetitive behaviors, and restricted interests. Although both genetic and environmental factors are involved, the underlying molecular mechanisms are unclear. Yong-Seok Lee at Seoul National University in South Korea and co-workers investigated the mechanisms underlying ASD using a drug-induced mouse model. They found that the ASD-model mice showed deficits in social behaviors and had higher levels of Rnf146 compared to control mice. Boosting levels of Rnf146 in control mice produced ASD-like behavior. Further investigation showed that the ASD-model mice had increased excitatory synaptic transmission in the prefrontal cortex, which regulates social behavior. These results indicate a promising target for ASD research.

## Introduction

Autism spectrum disorder (ASD) is a neurodevelopmental disorder with a range of behavioral manifestations, such as impaired social interaction and communication, repetitive behaviors, and restricted interests^1^. Studies have been carried out to map risk genes, the alterations of which are associated with ASD development, and those results have been accumulated in the SFARI gene database (https://gene.sfari.org)^2^. Aside from genetic factors, pre- and postnatal environmental factors also play crucial roles in the generation of ASD phenotypes, especially impaired social behavior^3,4^.

Valproic acid (VPA), or 2-propyl pentanoic acid, is a short-chain fatty acid clinically used as a mood stabilizer and antiepileptic drug^5^. VPA is a well-known inducer of neural tube defects and its prenatal exposure highly likely causes teratogenic effects that often lead to autistic-like behavior and developmental disorders^6^. A distinct linkage between prenatal exposure to VPA and autism has been confirmed^7–11^. Clinical studies have reported that in utero exposure to VPA is associated with an increased risk of impaired embryonic neurodevelopment and cognitive development and a higher incidence of ASD in offspring^12,13^. Rodents exposed to prenatal VPA (hereafter, VPA-exposed rodents) are widely considered animal models of ASD associated with prenatal environmental risk factors that allow investigation of its underlying neurobiological mechanism^11^. For example, VPA-exposed rodents showed autistic-like behaviors, such as decreased social interaction, repetitive behaviors, high pain sensitivity, and increased basal anxiety^14–16^. VPA-exposed rodents also exhibit alterations seen in other autism mouse models, including changes in brain connectivity, an excitatory/inhibitory imbalance, aberrant dendritic spine development in the hippocampus and cerebellar cortical formation, and deformation of Purkinje cells^16–21^. These findings provide further evidence that the model of prenatal VPA exposure has face validity at the cellular level.

Previous studies have proposed that the wingless (Wnt)/β-catenin signaling pathway, which is critical for brain development and synaptic functions, is one of the convergent cellular processes altered in ASD^22–24^. Exposure to VPA has also been shown to activate the Wnt and mTOR pathways^25^. Correspondingly, downregulating canonical Wnt/β-catenin signaling ameliorated ASD-like behavioral phenotypes in VPA-exposed rats^26^. VPA was also shown to increase the proliferation of neural progenitor cells by activating Wnt/β-catenin signaling during development^9^. However, the molecular mechanism underlying VPA-induced activation of the Wnt/β-catenin signaling pathway in the adult brain and the causality between Wnt/β-catenin signaling dysregulation and behavioral deficits are unclear.

Recent advances in omics technologies have enabled us not only to identify dysregulated molecules but also to gain a comprehensive understanding of the molecular mechanisms underlying ASD^27,28^. Beyond global gene expression profiling, single-cell genomics techniques have been employed to investigate the molecular pathophysiology of ASD in a cell type-specific manner^29^. Recently, mass spectrometry (MS)-based proteomic techniques, which allow us to directly study molecular networks in tissues or cells at the protein level^30,31^, have emerged as a valuable tool for identifying molecular changes associated with ASD in various model systems, including mice, humans, and forebrain organoids^28,32–34^. However, limited research has focused on identifying dysregulated proteins and signaling networks in VPA-exposed mice.

While the prefrontal cortex (PFC) is critical for regulating social behaviors and dysregulation of the PFC is often observed in humans with ASD as well as mouse models, including VPA-exposed rodents^35–37^, it is still unknown how prenatal VPA exposure affects the mouse PFC proteome, which may underlie ASD-like behavioral manifestations. Therefore, in this study, we employed a high-resolution mass spectrometry (MS)-based quantitative proteomic approach to identify differentially expressed proteins (DEPs) and altered signaling pathways in the PFC of VPA-exposed mice. We found that the expression of the ring-type E3 ubiquitin transferase Rnf146, which is a key regulator of the Wnt/β-catenin signaling pathway, is increased in the PFC of VPA-exposed mice. Furthermore, overexpression of Rnf146 in the PFC impairs excitatory synaptic transmission and social behavior in adult mice, demonstrating that Rnf146 upregulation contributes to social behavior deficits in a VPA-induced mouse model of ASD.

## Materials and methods

### Mice

CD1 (ICR) mice (CriOri; OrientBio, Korea) were used for VPA-exposed mouse preparation as previously described^38^. VPA (350 mg/kg, 10 ml/kg) or vehicle (0.9% saline) was subcutaneously injected into pregnant mice over the interscapular region at E10. Social behavior tests were performed 6-7 weeks after birth. Six-week-old C57BL/6N male mice (C57BL/6NCrljOri; OrientBio, Korea) were used for the prefrontal Rnf146 overexpression experiments. Rnf146- or eGFP-overexpressing mice were subjected to behavioral tests 4 weeks after viral injection. Animals were reared with a standard 12 h light/12 h dark cycle. All animal experiments were approved by the Institutional Animal Care and Use Committee of KonKuk University (KU18054) and Seoul National University (SNU-180521-3-6).

### Behavioral tests

Social behaviors were examined using the 3-chamber test as previously described^38^. Please see the supplementary methods for details regarding the behavioral tests.

### Tissue sampling

PFC samples were collected from VPA-treated or control mice one week after the behavioral tests and used for proteomics and western blot analyses. Brain tissues for western blotting were dissected from Rnf146- or eGFP-overexpressing mice 2–3 weeks after the behavioral tests. For RNA-seq, the PFC from Rnf146- or eGFP-overexpressing mice was dissected 4 weeks after viral injection. Mice were sacrificed under 5% isoflurane anesthesia. Brain tissues from mice were immediately dissected and frozen on dry ice and then stored at −80 °C until further use.

### Proteomic data generation and analysis

Quantitative tandem mass spectrometry data were acquired as previously described^28,39^. Briefly, PFC proteins were extracted from VPA-exposed (*n* = 4) and control mice (*n* = 4) and were enzymatically digested by sequencing-grade trypsin. Peptides were chemically labeled using TMT reagents, and pooled TMT-barcoded peptides were fractionated by high pH reversed-phase liquid chromatography. A peptide mixture in each fraction was analyzed with a high-resolution Orbitrap mass spectrometer (Thermo Scientific, USA) in data-dependent acquisition mode.

The tandem mass spectra obtained were analyzed with MaxQuant software as previously described^40^. Identified peptides and proteins were normalized to carry out principal component analysis, multiscatter plot analysis, and hierarchical clustering analysis. Please see the supplementary methods and Supplementary Fig. 2 for more details regarding proteomic data generation and analysis.

### Enrichment analyses of DEPs

Proteins with significant differences in expression in VPA-exposed mice compared to control mice (*p* value ≤ 0.05) were considered VPA-DEPs. Using the homolog database from Mouse Genome Informatics (http://www.informatics.jax.org/homology.shtml), we converted the mouse protein list into a list of human homologs; mouse proteins without human homologs were excluded from the analysis. Please see the supplementary methods for a detailed description of the enrichment analyses.

### Functional mapping and protein‒protein interaction (PPI) network

We evaluated the biological functions of the VPA-DEPs in terms of the pathways from the MsigDB Gene Ontology and Reactome Database^41^ using the EnrichR tool^42^. Please see the supplementary methods for a detailed description of the functional mapping and PPI network.

### RNA sequencing of DEGs in Rnf146-overexpressing mice

Raw reads were processed with Cutadapt^43^ for Illumina adapter trimming and removal of low-quality reads. We removed 15 bp of each read for low quality (*q* < 30) and discarded reads shorter than 100 base pairs. After a quality check with FastQC (http://www.bioinformatics.babraham.ac.uk/projects/fastqc/), we quantified the abundance of transcripts using Salmon software (v1.9.0)^44^ in quasi-mapping-based mode referring to the mouse reference transcriptome (GENCODE vM30, GRCm39). Please see the supplementary methods for details.

### WGCNA network construction and module identification

To identify the functional topology in samples, weighted gene coexpression network analysis (WGCNA, version 1.71) was applied to transcriptomics data. Please see the supplementary methods for details.

### Viral vector construction and AAV packaging

An adeno-associated viral (AAV) vector for neuronal expression of Rnf146 (pAAV-hsyn-mRnf146-T2A-eGFP-WPRE) was constructed by recombinant PCR. The AAV was prepared as previously described^45^. Please see the supplementary methods for a detailed description of vector construction and AAV packaging.

### Surgical procedures

Surgical procedures were performed as previously described^46^. After anesthetization with a mixture of Zoletil (30 mg/kg) and Rompun (10 mg/kg) via i.p. injection, mice were placed on the rodent stereotaxic apparatus. AAV (5 × 10^12^ GC/mL, 200 nL in ACSF) was bilaterally injected into the PFC (AP + 2.4 from bregma, ML ± 0.5 from midline, DV −2.4 and −1.8 from skull) using a Nanolitre 2010 (WPI, USA) at a speed of 20 μL/min. Animals were given at least 3 weeks of rest before further experiments.

### Western blot analysis

Total protein lysates (10 µg) were separated on a 10% gel by SDS‒PAGE and transferred to a nitrocellulose membrane (Bio-Rad, USA). After blocking in 5% skim milk or BSA, membranes were incubated with primary antibodies (phospho-β-catenin, #9561, Cell Signaling; β-catenin, #9582, Cell Signaling; RNF146, NBP1-85318, NOVUS; α-Tubulin, sc-8035, Santa Cruz) overnight at 4 °C. After incubation with appropriate HRP-conjugated secondary antibodies, chemiluminescent signals were detected by AI600 (Amersham™, USA) using ECL solution (32209; Pierce, USA).

### Statistical analysis

A two-tailed unpaired t test was used for the statistical comparisons of the preference index (PI) and protein expression levels of VPA- and vehicle-exposed mice. Statistical analyses for behavioral tests and western blot analyses were carried out using GraphPad Prism v7 software. All data are shown as the mean ± SEM.

## Results

### Social behavior deficits in VPA-exposed mice

**Fig. 1:**
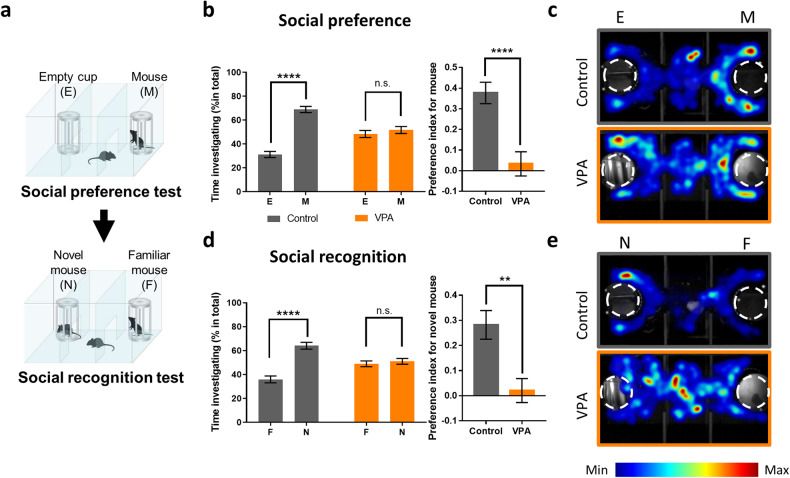
Social deficits in VPA-exposed mice. **a** Schematic diagram of the 3-chamber social behavior test. The social preference and recognition of vehicle (control)- or VPA-exposed mice were evaluated. **b** Left: in the social preference test, VPA-exposed mice did not show a significant preference for a novel conspecific (M) over an empty cup (E), while control mice showed a significant preference for the novel conspecific (*n* = 19 mice for control, *n* = 20 mice for VPA, two-way ANOVA: VPA, *F*_1, 74_ = 2.290e−014, *P* > 0.9999; target, *F*_1, 74_ = 54.32, ****P < 0.0001; interaction, *F*_1, 74_ = 38.2, ****P < 0.0001; Sidak’s multiple comparisons test: control, mouse versus object *t*_74_ = 9.461, ****P < 0.0001; VPA, mouse versus object *t*_74_ = 0.8525, *P* = 0.6360). Right: preference index (PI) of control and VPA-exposed mice. VPA-exposed mice showed significantly lower PI values than control mice (*n* = 19 for control, *n* = 20 for VPA, two-tailed unpaired *t* test; *t*_37_ = 4.37, ****P < 0.0001). **c** Representative heatmap images of the social preference test of control (upper) and VPA-exposed mice (lower). **d** Left: in the social recognition test, VPA-exposed mice did not show a significant preference for a novel conspecific (N) versus a familiar conspecific (F), while control mice showed a significant preference for the novel conspecific (*n* = 19 mice for control, *n* = 20 mice for VPA, two-way ANOVA: VPA, *F*_1, 74_ = 0.000, *P* > 0.9999; target, *F*_1, 74_ = 33.36, ****P < 0.0001; interaction, *F*_1, 74_ = 24.82, ****P < 0.0001; Sidak’s multiple comparisons test: control, novel versus familiar *t*_74_ = 7.511, ****P < 0.0001; VPA, novel versus familiar *t*_74_ = 0.5683, *P* = 0.8164). Right: PI of control and VPA-exposed mice. VPA-exposed mice showed significantly lower PI values than control mice (*n* = 19 for control, *n* = 20 for VPA, two-tailed unpaired *t* test; *t*_37_ = 4.523, **P = 0.0012). **e** Representative heatmap images of the social recognition test of control mice (upper) and VPA-exposed mice (lower).

Studies have established that in utero exposure to VPA induces social behavior deficits in offspring^14,38^. The three-chamber test has been widely used to examine social behaviors in mice^47^. Since mice are social animals, they prefer to spend more time interacting with a conspecific than exploring an object or empty cup in the three-chamber test (Fig. 1a)^47^. We confirmed that VPA-exposed mice did not show a preference for the conspecific over the empty cup and subsequently showed a significantly lower social preference index than control mice (Fig. 1b, c). In addition, VPA-exposed mice did not show a preference for a novel conspecific over a familiar conspecific, while control mice showed a significant preference for the novel conspecific (Fig. 1d, e). In addition, we confirmed that in utero exposure to VPA induces teratogenic effects. We observed that VPA-exposed dams gave birth to significantly smaller litters than control dams (Supplementary Fig. 1a), and most VPA-exposed offspring exhibited a crooked tail phenotype resulting from neural tube defects (Supplementary Fig. 1b, c)^6^. Notably, we found a significant correlation between the social preference index and the angles of the crook point in VPA-exposed mouse tails (Supplementary Fig. 1d), providing further evidence that prenatal VPA exposure induces developmental deficits in offspring. After evaluating these behavioral and morphological phenotypes, the prefrontal cortex (PFC) of VPA- and vehicle-exposed mice was dissected for proteomic analysis.

### Proteomic analysis in VPA-exposed mice

**Fig. 2:**
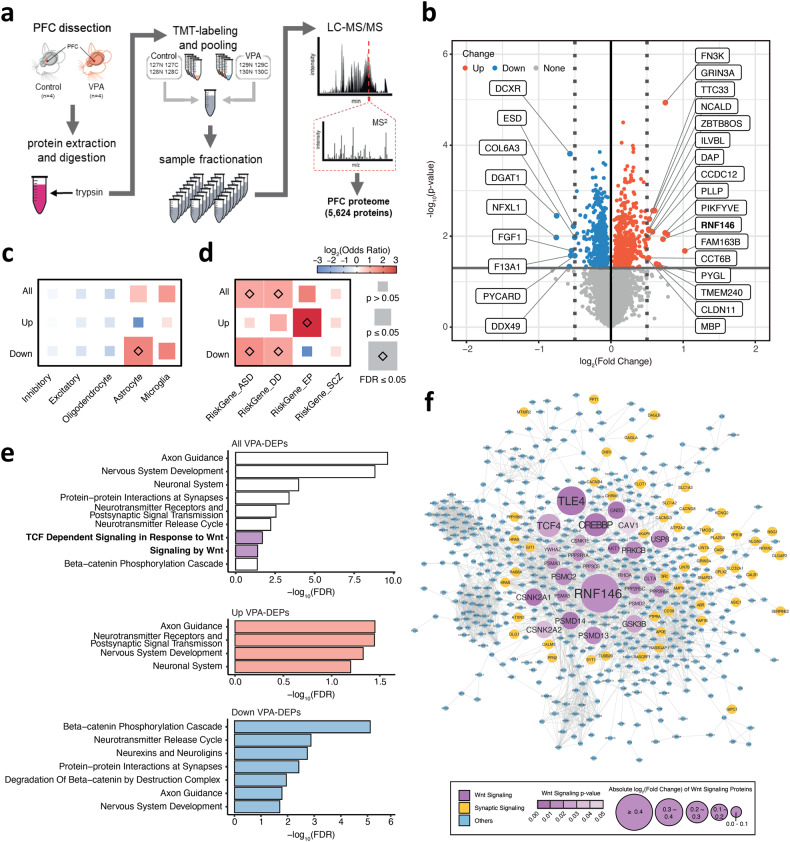
VPA-exposed mice show upregulation of the Wnt signaling pathway. **a** The overall design of the proteomic experiment. The dissected PFC tissues were homogenized and sonicated, and tryptic digestion was performed to break proteins down into peptides, which were further labeled with TMT isobaric labeling of 8 samples for 24 fractionations, followed by mass spectrometry quantitative analysis. **b** Volcano plot showing differentially expressed proteins between VPA-exposed mice and control mice (VPA-DEPs). The *x*-axis represents the log_2_ scale of the fold change in differential expression, while the *y*-axis represents the −log_10_ scale of the significance in changes. VPA-DEPs exhibiting expression changes with an absolute log_2_-fold change equal to or greater than 0.05 are labeled in the plot. **c** Enrichment analysis of VPA-DEPs with genes specific to five distinct cell types, as defined by single-cell RNA-seq data from the mouse nervous system. The log_2_(odds ratio) values that exceeded the range of −3 to 3 were adjusted to fit within this range. The blue and red colors represent the log_2_(odds ratio) values, and the size of the box corresponds to the significance in enrichment. **d** Enrichment analysis of VPA-DEPs with disorder risk genes. The log_2_(odds ratio) values that exceeded the range of −3 to 3 were adjusted to fit within this range. The blue and red colors represent the log_2_(odds ratio) values, and the size of the box corresponds to the significance in enrichment. Autism spectrum disorder (ASD), developmental disorder (DD), epilepsy (EP), and schizophrenia (SCZ). **e** Functional annotations for VPA-DEPs showing significantly enriched biological pathways (FDR ≤ 0.05). The x-axis represents the −log_10_ scale of significance in enrichment. The purple highlighted bars indicate the pathway associated with Wnt signaling enriched with all VPA-DEPs. The enriched pathways for upregulated and downregulated VPA-DEPs are highlighted in red and blue, respectively. **f** Protein–protein interaction (PPI) network of VPA-DEPs revealing proteins related to Wnt signaling and neuronal development.

To characterize the effects of VPA on molecular signaling, we conducted a TMT-based high-throughput quantitative proteomic analysis using a high-resolution Orbitrap mass spectrometer (Fig. 2a). A total of 5624 proteins were quantitatively identified with ~17.5% median sequence coverage (Fig. 2a and Supplementary Fig. 2a). After quantile normalization (Supplementary Fig. 2b), we carried out principal component analysis, which showed a distinct separation between VPA-exposed and control brain tissues (Supplementary Fig. 2c). Pearson correlation coefficients between these samples had the lowest value of 98.7%, demonstrating good reproducibility between biological replicates (Supplementary Fig. 2d). The hierarchical clustering of DEPs was distinct, as shown in a heatmap visualizing the differences in protein expression (Supplementary Fig. 2e).

Among the 5624 identified proteins, 941 proteins were identified as DEPs in VPA-exposed mice (VPA-DEPs) compared to controls, including 460 upregulated and 481 downregulated DEPs (Fig. 2b, Supplementary Table 1a). To determine the cellular specificity of VPA-DEPs for the mouse nervous system, we conducted an enrichment test using cell-type marker genes across the central nervous system (Fig. 2c, Supplementary Table 1b)^48^. We found that downregulated VPA-DEPs were significantly enriched in astrocyte marker genes (odds ratio [OR] = 3.6, false discovery rate [FDR] = 1.9 × 10^−4^, one-sided Fisher’s exact test [FET]). Furthermore, these downregulated VPA-DEPs showed significant overlap with astrocytes in the human brain when compared with cell-type marker genes for the developing human brain cortex (OR = 3.7, FDR = 9.0 × 10^−6^, one-sided FET; Supplementary Fig. 3, Supplementary Table 1c)^49^. Taken together, these findings suggest potential decreases in the expression of astrocyte-specific proteins in the PFC by in utero VPA exposure.

We further evaluated the enrichment of VPA-DEPs with differentially expressed genes (DEGs) in the postmortem cortex of ASD patients. From Parikshak et al.^50^, we used two gene sets: (1) up- or downregulated DEGs enriched in ASD brains compared to control brains and (2) coexpressed network genes of 25 modules. We found a significant enrichment of downregulated VPA-DEPs in the cortex. M9 (OR = 2.4, FDR = 2.6 × 10^−3^, one-sided FET) (Supplementary Fig. 4, Supplementary Table 1d), which was an astrocyte-specific module associated with ASD brains. We further assessed whether VPA exposure alters the expression of known ASD or neurodevelopmental disorder (NDD) risk genes. To do this, we collated a list of high-confidence risk genes for ASD, developmental delay (DD), epilepsy (EP), and schizophrenia (SCZ), prioritized based on large-scale exome studies^51–54^. Notably, we found that downregulated VPA-DEPs were significantly enriched in ASD risk genes (OR = 3.0, FDR = 8.4 × 10^−3^, one-sided FET) and DD risk genes (OR = 2.4, FDR = 2.7 × 10^−2^, one-sided FET) (Fig. 2d, Supplementary Table 1e). Among them, several genes have been implicated in the Wnt/β-catenin signaling pathway. *Crebbp* is part of a transcriptional regulatory complex inhibiting the Wnt/β-catenin signaling pathway^55^. Synaptotagmin-1 (Syt1) was reported to be the Wnt target molecule for neurotransmitter release at excitatory synapses^56^. In addition, ubiquitin-specific peptidase 9 X-linked (USP9X) was shown to regulate the canonical and noncanonical Wnt pathways in cancer development via the deubiquitylation of DVL2^57^. We also compared our findings with previous proteomics studies using other ASD mouse models, such as *Cntnap2*, *Elp2*, and *Pten* mutant mice (Supplementary Fig. 5, Supplementary Table 1f)^28,33,34^. The downregulated VPA-DEPs in our study showed significant overlap with upregulated DEPs in other ASD proteomics studies, primarily involving proteins associated with the neurodegeneration pathway (KEGG: hsa05022, OR = 15.5, *p*-value = 1.9 × 10^−3^).

We performed functional annotations and identified significant enrichment of VPA-DEPs in Wnt/β-catenin signaling-related pathways (Fig. 2e, Supplementary Table 1g–i). VPA-DEPs were significantly enriched for signaling by WNT (R-HSA-195721, OR = 1.8, FDR = 4.0 × 10^−2^) and TCF-dependent signaling in response to WNT (R-HSA-201681, OR = 2.2, FDR = 2.0 × 10^−2^). In particular, downregulated VPA-DEPs were significantly enriched for β-catenin-related pathways, including the beta-catenin phosphorylation cascade (R-HSA-196299, OR = 2.4, FDR = 7.8 × 10^−6^) and degradation of beta-catenin by the destruction complex (R-HSA-195253, OR = 7.9, FDR = 1.1 × 10^−2^). Among these pathway genes, the largest perturbation of protein expression was observed for RNF146 (log_2_(fold change) = 0.72; *p*-value = 1.2 × 10^−2^), which has been suggested to be a key regulator of Wnt/β-catenin signaling (Fig. 2f). This result implies that the upregulation of RNF146 mediates the VPA-induced modulation of the Wnt signaling pathway.

### Verification of RNF146 upregulation in the PFC of VPA-exposed mice

**Fig. 3:**
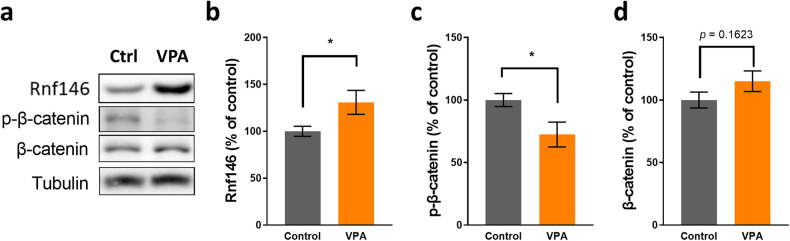
VPA-exposed mice show increased Rnf146 expression. **a** Representative western blot results. Ctrl, vehicle-exposed control mice; VPA, VPA-exposed mice. **b** VPA-exposed mice showed significantly increased Rnf146 levels compared to control mice (*n* = 14 mice for control, *n* = 13 mice for VPA, two-tailed unpaired *t* test; *t*_25_ = 2.283, *P = 0.0312). Rnf146 expression levels were normalized to tubulin levels. **c** VPA-exposed mice showed significantly decreased p-β-catenin levels, which were normalized to β-catenin levels, compared to control mice (*n* = 14 mice for control, *n* = 13 mice for VPA, two-tailed unpaired *t* test; *t*_25_ = 2.519, *P = 0.0185). **d** VPA-exposed mice showed comparable β-catenin levels, which were normalized to tubulin levels, when compared to control mice (*n* = 14 mice for control, *n* = 13 mice for VPA, two-tailed unpaired *t* test; *t*_25_ = 1.456, *P* = 0.1577).

To verify the proteomics results of alterations in Wnt/β-catenin signaling, we examined the protein expression level of a selected target protein, Rnf146. Consistent with the proteomics data, the protein expression level of Rnf146 was significantly increased in the PFC of VPA-exposed mice compared to that in the vehicle-exposed control mice (Fig. 3a, b). Rnf146 positively regulates the Wnt/β-catenin pathway by inducing the degradation of Axin, which is the concentration-limiting component of β-catenin^58^. Axin forms a multiprotein β-catenin destruction complex with GSK3 and adenomatous polyposis coli (APC), which induces the phosphorylation and subsequent degradation of β-catenin^59^. Consistent with these findings, it has been shown that the activity of Wnt/β-catenin signaling is elevated in VPA-exposed mice^9^. Moreover, in the present experiment, phosphorylated β-catenin (p-β-catenin) was significantly decreased in the PFC of VPA-exposed mice compared with the control group, demonstrating alterations in the Rnf146-Wnt/β-catenin signaling pathway in VPA-exposed mice (Fig. 3c). However, while there was a mild trend of increase in β-catenin levels in VPA-exposed mice compared to control mice, the total amount of β-catenin was not significantly different between the two groups (Fig. 3d).

### Social behavior deficits in RNF146-overexpressing mice

**Fig. 4:**
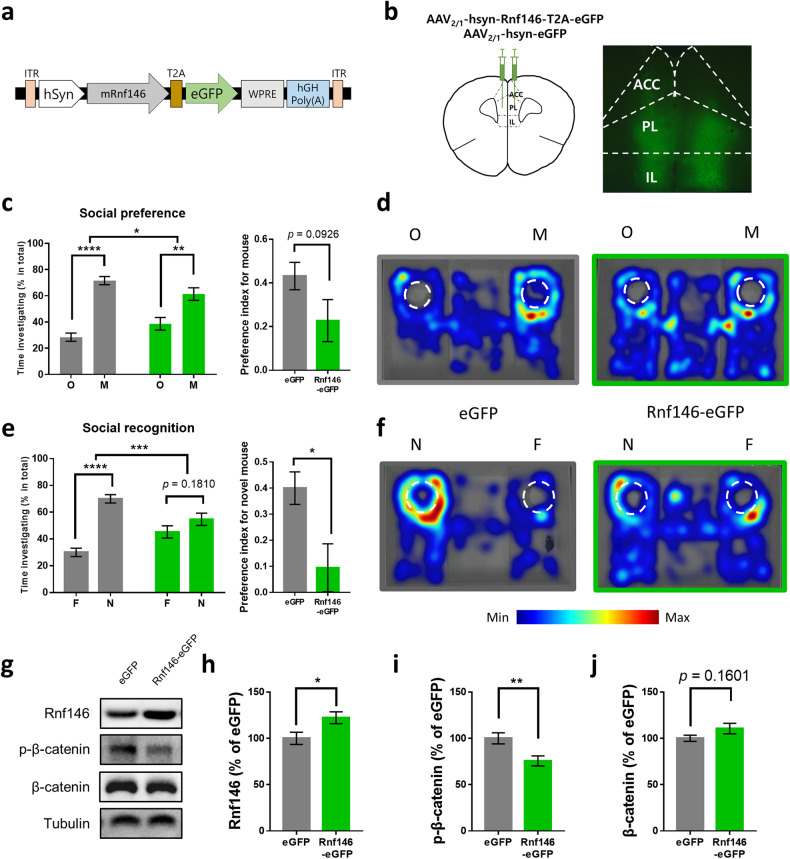
Rnf146 overexpression in the PFC causes social deficits in mice. **a** Diagram for AAV constructs encoding mouse Rnf146 (mRNF146) and eGFP. ITR, inverted terminal repeat sequence; WPRE, Woodchuck hepatitis virus (WHV) posttranscriptional regulatory element. **b** Schematic diagram of bilateral AAV virus injection in the PFC (left) and an example of a brain slice expressing Rnf146 in the PFC (right). ACC, anterior cingulate cortex; PL, prelimbic cortex; IL, infralimbic cortex. **c** Left: In the social preference test, Rnf146-overexpressing mice showed a significant preference for a novel conspecific (M) versus an object (O) compared to eGFP-overexpressing control mice (*n* = 11 mice for eGFP, *n* = 11 mice for Rnf146, two-way ANOVA: Rnf146, *F*_1, 40_ = 0.000, *P* > 0.9999; target, *F*_1, 40_ = 65.87, ****P < 0.0001; interaction, *F*_1, 20_ = 6.025, *P = 0.0234; Sidak’s multiple comparisons test: eGFP, mouse versus object *t*_40_ = 7.52, ****P < 0.0001; Rnf146, mouse versus object *t*_40_ = 3.958, ***P = 0.0006). Right: preference index (PI) of eGFP- or Rnf146-overexpressing mice. Rnf146-overexpressing mice showed PI values comparable to those of control mice (*n* = 11 mice for eGFP, *n* = 11 mice for Rnf146, two-tailed unpaired *t* test; *t*_20_ = 1.781, *P* = 0.0901). **d** Representative heatmap images from eGFP-overexpressing mice (left) and Rnf146-overexpressing mice (right) of social behavior during the social preference test. **e** Left: in the social recognition test, Rnf146-overexpressing mice did not show a significant preference for a novel conspecific (N) versus a familiar conspecific (F), while eGFP-overexpressing control mice showed a significant preference for the novel conspecific (*n* = 11 mice for eGFP, *n* = 11 mice for Rnf146, two-way ANOVA: Rnf146, *F*_1, 40_ = 0, *P* > 0.9999; target, *F*_1, 40_ = 39.94, ****P < 0.0001; interaction, *F*_1, 40_ = 15.22, ***P = 0.0004; Sidak’s multiple comparisons test: eGFP, novel versus familiar *t*_40_ = 7.227, ****P < 0.0001; Rnf146, novel versus familiar *t*_40_ = 1.71, *P* = 0.1810). Right: PI of eGFP- or Rnf146-overexpressing mice. Rnf146-overexpressing mice showed significantly lower PI values than control mice (*n* = 11 mice for eGFP, *n* = 11 mice for Rnf146 group, two-tailed unpaired *t* test; *t*_20_ = 2.759, *P = 0.0121). **f** Representative heatmap images from eGFP-overexpressing mice (left) and Rnf146-overexpressing mice (right) of social behavior during the social recognition test. **g** Western blot analysis of the Wnt signaling pathway in the PFC of Rnf146-overexpressing mice. Samples from the PFC of GFP-overexpressing mice were used as controls. **h** Rnf146-overexpressing mice showed significantly increased Rnf146 levels (normalized to tubulin levels) compared to eGFP-overexpressing mice (*n* = 7 mice for eGFP, *n* = 10 mice for Rnf146, two-tailed unpaired *t* test; *t*_14_ = 2.381, *P = 0.0320). **i** Rnf146-overexpressing mice showed significantly decreased p-β-catenin levels (normalized to β-catenin levels) compared to eGFP-overexpressing mice (*n* = 7 mice for eGFP, *n* = 10 mice for Rnf146, two-tailed unpaired *t* test; *t*_14_ = 2.995, **P = 0.0096). **j** Rnf146-overexpressing mice showed a comparable β-catenin level (normalized to tubulin levels) compared to eGFP-overexpressing mice (*n* = 7 mice for eGFP, *n* = 10 mice for Rnf146, two-tailed unpaired *t* test; *t*_14_ = 1.483, *P* = 0.1601).

To examine whether the elevated expression of Rnf146 is associated with social behavior deficits in VPA-exposed mice, we overexpressed Rnf146 in the PFC of naïve C57BL/6 mice using an AAV (Fig. 4a, b). In the social preference test, both Rnf146- and eGFP-overexpressing mice spent significantly more time exploring a conspecific than an object, while the Rnf146-expressing mice showed a weaker social preference than GFP-expressing mice (Fig. 4c, d). Interestingly, however, Rnf146-overexpressing mice showed a deficit in the social recognition test, as they did not show a preference for a novel conspecific over a familiar conspecific, while control eGFP-overexpressing mice showed a significant preference for a novel conspecific (Figs. 4e, f). These results demonstrate that Rnf146 overexpression caused a severe deficit in social recognition with a mild impact on social preference in mice. Consistent with the results from VPA-exposed mice, the level of p-β-catenin was significantly decreased in the PFC of Rnf146-overexpressing mice compared with the control group, and the amount of total β-catenin tended to increase in Rnf146-overexpressing mice compared to that in eGFP-overexpressing mice (Fig. 4g–j). Rnf146-overexpressing mice showed basal locomotor activity and anxiety levels comparable to those of eGFP-overexpressing control mice (Supplementary Fig. 6).

### Altered Wnt/β-catenin signaling pathway in RNF146-overexpressing PFC

**Fig. 5:**
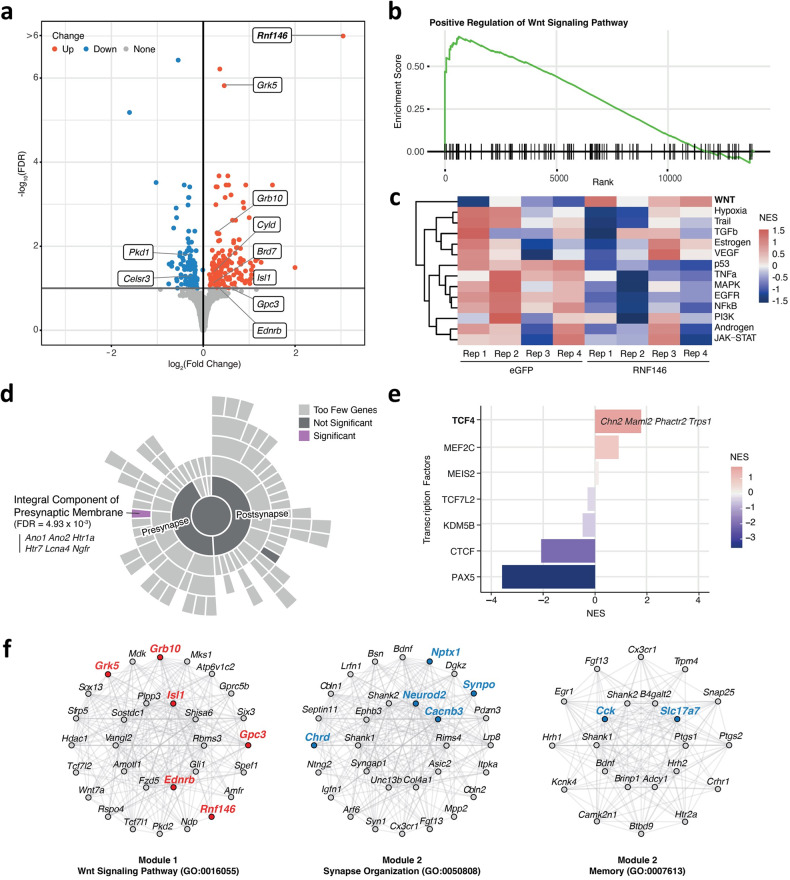
The Wnt signaling pathway is promoted by Rnf146 overexpression. **a** Volcano plot showing differentially expressed genes between Rnf146-overexpressing mice and eGFP-overexpressing mice (Rnf146-DEGs). The *x*-axis represents the log_2_ scale of the fold change in differential expression, while the *y*-axis represents the -log_10_ scale of the significance changes. Rnf146-DEGs associated with the Wnt signaling pathway are labeled in the plot. **b** Gene set enrichment plot depicting the elevated positive regulation of the Wnt signaling pathway in Rnf146-overexpressing mice. **c** Heatmap of the pathway activity for each sample inferred by PROGENy. The levels of pathway activity reflect the extent of pathway deregulation. **d** Enrichment analysis of the upregulated Rnf146-DEGs with pathway terms describing synaptic locations in the brain. Pathway analysis was performed using one-sided Fisher’s exact test to identify significantly overrepresented terms. The upregulated Rnf146-DEGs significantly overlapped (FDR < 0.01) with the integral component of the presynaptic membrane pathway (purple). Pathway terms with two or fewer overlapping genes were excluded and indicated as too few genes. **e** Enrichment analysis of Rnf146-DEGs with the inferred activity of transcription factors associated with ASD. Upregulated target genes of the transcription factor TCF4 are annotated in the plot. NES, normalized enrichment score. **f** Network of genes in WGCNA module 1 enriched in the Wnt signaling pathway and genes in module 2 enriched in synapse organization and memory. In each network, the top 30 hub genes (22 for memory) with the highest levels of intramodular connectivity were depicted. Rnf146-DEGs are highlighted in bold, with red and blue colors indicating up- and downregulation, respectively.

To investigate whether Rnf146 overexpression induces transcriptional changes in the PFC through Wnt/β-catenin signaling activation and to elucidate the molecular mechanism underlying the social deficits induced by Rnf146 overexpression, we conducted RNA sequencing analysis using mice that were confirmed to overexpress Rnf146 and exhibit social deficits. When compared with eGFP-overexpressing mice, Rnf146-overexpressing mice exhibited a total of 294 DEGs (Rnf146-DEGs) (FDR ≤ 0.1), some of which overlapped with VPA-DEPs (*n* = 11, *Adgrb2, Chrm1, Fmnl1, Islr2, Mtmr2, Plppr1, Ripor2, Rnf146, Smpd4, Tmem132a*, and *Trir*) (Fig. 5a and Supplementary Table 2a). Importantly, Rnf146 overexpression resulted in a significant enrichment with positive regulation of Wnt signaling pathways (GO:0030177; normalized enrichment score (NES) = 1.7, FDR = 4.9 × 10^−2^) (Fig. 5b and Supplementary Table 2b). Along with *Rnf146*, the Rnf146-DEGs that were mainly enriched in this pathway included *Gpc3*, which regulates the interaction between Wnt and Frizzled and leads to the activation of downstream signaling pathways^60^. We further calculated the pathway activity scores of 14 signaling processes in each sample. Rnf146-overexpressing mice showed elevated Wnt signaling pathway (NES = 1.5, *p*-value = 1.2 × 10^−2^) (Fig. 5c and Supplementary Table 2c). These results indicate that Rnf146 plays a pivotal role in modulating Wnt/β-catenin signaling in the mouse PFC.

To further explore whether Rnf146 overexpression alters the expression of synaptic molecules that may be associated with behavioral deficits, we analyzed 266 pathway terms of synaptic locations and processes from the SynGO database^61^. The upregulated Rnf146-DEGs (*Ano1, Ano2, Htr1a, Htr7, Kcna4*, and *Ngfr*) significantly overlapped with an integral component of the presynaptic membrane (GO:0099056, FDR = 4.9 × 10^−3^) (Fig. 5d, Supplementary Table 2d). As Wnt is known to regulate presynaptic assembly and neurotransmitter release across synapses, this finding supports the role of Rnf146 in Wnt/β-catenin signaling^62^. Moreover, regarding the transcription factors linked to ASD, Rnf146 overexpression significantly increased the activity of the transcription factor TCF4, which was accompanied by an upregulation of TCF4 target genes such as *Chn2, Maml2, Phactr2* and *Trps1* (NES = 1.8, *p*-value = 3.66 × 10^−3^) (Fig. 5e, Supplementary Table 2e). TCF4 binds to β-catenin and transactivates Wnt target genes, indicating that TCF4 could be the primary target of Rnf146 overexpression for activating the Wnt/β-catenin signaling pathway^63^. Overall, these findings suggest that Rnf146 plays a crucial role in regulating the Wnt/β-catenin signaling pathway in the PFC of mice.

To delineate the core gene network of Rnf146-overexpressing mice, we analyzed gene coexpression using Rnf146- and eGFP-overexpressing transcriptomics datasets. We found 51 coexpression modules with ~200 to 1200 genes (Fig. 5f and Supplementary Fig. 7, Supplementary Tables 2f, 2g, and 2h), representing biological pathways such as nervous system development (Module 1), synapse organization, and memory (Module 2). Module 1 was characterized by the Wnt signaling pathway (GO:0016055, FDR = 4.8 × 10^−3^), in which *Rnf146* and its interacting genes (*n* = 36) were coexpressed. This module was significantly enriched in upregulated Rnf146-DEGs, implicating the core genes perturbed by Rnf146 overexpression (OR = 47.3, FDR = 9.2 × 10^−108^, one-sided FET). In contrast, Module 2, which was characterized by pathways associated with synapse organization (GO:0050808, FDR = 4.0 × 10^−8^) and memory (GO:0007613, FDR = 3.1 × 10^−5^), was significantly enriched in downregulated Rnf146-DEGs, including *Cacnb3*, *Cck*, *Chrd*, *Neurod2*, *Nptx1*, *Slc17a7*, and *Synpo*. Coexpressed genes of Module 2 also included *Ube3b*, which was significantly perturbed by Rnf146 overexpression and highly associated with intellectual disability (eigengene-based connectivity = 0.96) (Supplementary Table 2f)^64,65^.

### Increased excitatory synaptic transmission in RNF146-overexpressing neurons

**Fig. 6:**
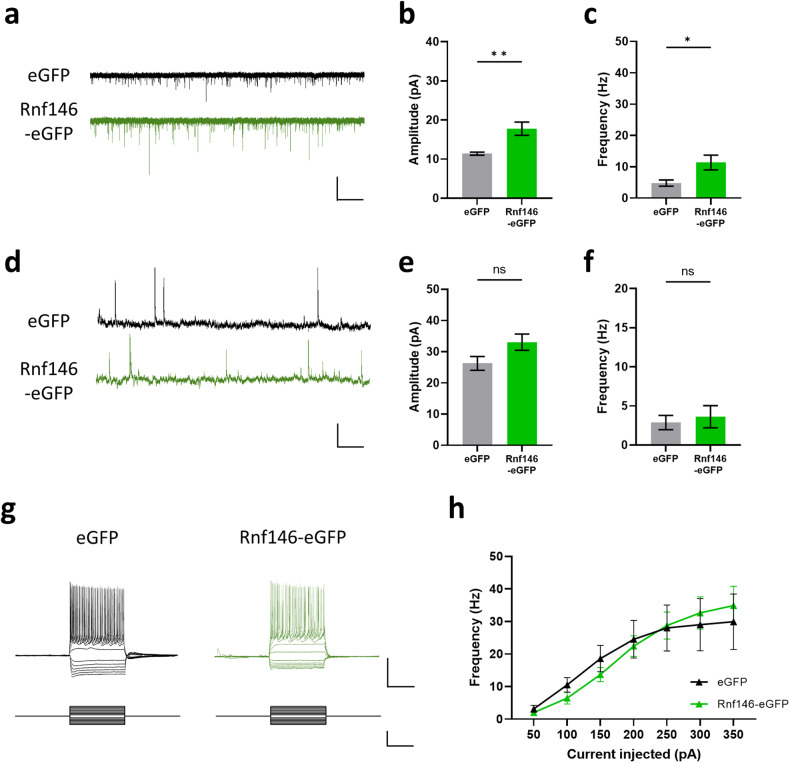
Rnf146 overexpression increases excitatory synaptic transmission in prefrontal pyramidal neurons. **a** Spontaneous excitatory postsynaptic current (sEPSC) traces of prefrontal neurons in Rnf146- and eGFP-overexpressing mice. Scale bar: 50 pA, 500 ms. **b** The sEPSC amplitude of prefrontal neurons in Rnf146-overexpressing mice was significantly larger than that in eGFP-overexpressing mice (n = 13 cells from 5 mice for eGFP, *n* = 16 cells from 6 mice for Rnf146; two-tailed unpaired *t* test, *t*_*27*_ = 3.302, **P = 0.0027). **c** The sEPSC frequency of prefrontal neurons in Rnf146-overexpressing mice was significantly larger than that in eGFP-overexpressing mice (*n* = 13 cells from 5 mice for eGFP, *n* = 16 cells from 6 mice for Rnf146; two-tailed unpaired *t* test, *t*_*27*_ = 2.373, *P = 0.0250). **d** Spontaneous inhibitory postsynaptic current (sIPSC) traces of prefrontal neurons in Rnf146- and eGFP-overexpressing mice. Scale bar: 100 pA, 500 ms. **e** The sIPSC amplitude of prefrontal neurons in Rnf146-overexpressing mice is comparable to that in eGFP-overexpressing mice (*n* = 11 cells from 4 mice for eGFP, *n* = 12 cells from 4 mice for Rnf146; two-tailed unpaired *t* test, *t*_*21*_ = 1.980, *P* = 0.0609). **f** The sIPSC frequency of prefrontal neurons in Rnf146-overexpressing mice is comparable to that in eGFP-overexpressing mice (*n* = 11 cells from 4 mice for eGFP, *n* = 12 cells from 4 mice for Rnf146; two-tailed unpaired *t* test, *t*_*21*_ = 0.4323, *P* = 0.6699). **g** Representative traces of voltage responses of prefrontal neurons in Rnf146- or eGFP-overexpressing mice evoked by current step stimuli (−300-300 pA, 50 pA increment, 1 s). Scale bar: upper, 50 mV, 500 ms; lower, 500 pA, 500 ms. **h** Summary data of the number of action potentials evoked in response to 300 pA current steps. The number of AP spikes was comparable in the prefrontal neurons of Rnf146-overexpressing mice relative to those of eGFP-overexpressing mice (*n* = 13 cells from 5 mice for eGFP, *n* = 16 cells from 5 mice for Rnf146; two-way ANOVA with repeated measures: effect of injected current, *F*_6, 162_ = 28.24, ****P < 0.0001; effect of Rnf146 expression, *F*_1, 27_ = 0.006249, *P* = 0.9376; interaction between current injection and Rnf146 expression, *F*_6, 162_ = 0.7368, *P* = 0.6207, Sidak’s multiple comparison test for eGFP versus Rnf146. 50 pA, *t*_*189*_ = 0.1724, *P* > 0.9999; 100 pA, *t*_*189*_ = 0.6127, *P* = 0.9957; 150 pA, *t*_*189*_ = 0.7362, *P* = 0.9870; 200 pA, *t*_*189*_ = 0.3139, *P* > 0.9999; 250 pA, *t*_*189*_ = 0.1120, *P* > 0.9999; 300 pA, *t*_*189*_ = 0.5416, *P* = 0.9980; 350 pA, *t*_*189*_ = 0.7398, *P* = 0.9867).

To further investigate the neuronal mechanism underlying impaired social behaviors in Rnf146-overexpressing mice, we performed whole-cell patch-clamp recordings. Previous studies have shown that electrophysiological properties are altered in the PFC of VPA-exposed mice^38,66,67^. We found that both the amplitude and frequency of spontaneous excitatory postsynaptic currents (sEPSCs) were significantly increased in Rnf146-expressing neurons compared to eGFP-expressing control neurons (Fig. 6a–c), whereas there was no significant change in spontaneous inhibitory postsynaptic currents (sIPSCs) (Fig. 6d–f). Finally, Rnf146 overexpression did not affect neuronal excitability (Fig. 6g, h) or other electrophysiological properties (Supplementary Table 3). These data suggest that Rnf146 overexpression may cause social behavior deficits by altering the excitation/inhibition balance in neurons of the PFC.

## Discussion

ASD can be caused by environmental factors, including prenatal drug exposure. One of the most widely used mouse models of ASD is prenatal VPA exposure; these mice exhibit an ASD-like phenotype, including social deficits. Despite several proteomic studies in a similar context, no study has reported quantitative proteomic analysis of the PFC of the VPA-exposed mouse model of ASD. Here, we utilized a TMT-based quantitative proteomic approach to identify novel molecular pathways underlying chemical-induced ASD pathophysiology. We identified Rnf146 as a hub protein responsible for the activation of the Wnt/β-catenin signaling pathway in VPA-exposed mice. Rnf146 overexpression in naïve adult mice impaired social behaviors, which may be caused by increased excitatory synaptic transmission. Consistent with these findings, our transcriptome analysis of the PFC of Rnf146-overexpressing mice revealed alterations in the Wnt signaling pathway. This study showed, for the first time, that Rnf146 plays a key role in modulating social behavior.

We found a significant overlap between DEPs in the PFC of VPA-exposed mice (VPA-DEPs) and DEGs in the postmortem cortex of patients with ASD. Additionally, the downregulated VPA-DEPs were significantly enriched in previously identified ASD and DD risk alleles. Moreover, when comparing our proteomics data with other ASD mouse models, such as *Cntnap2*, *Elp2*, and *Pten* mutant mice, we observed significant overlaps among DEPs. However, it is interesting that downregulated VPA-DEPs in our study showed significant overlap with upregulated DEPs in other ASD models^28,33,34^. This suggests an opposite direction of pathophysiology among ASD mouse models and a potentially distinct pathway in VPA-exposed mice. Importantly, only VPA-exposed mice showed Rnf146 as a DEP.

Rnf146 is an E3 ubiquitin-protein ligase that prevents the formation of the β-catenin destruction complex by inducing Axin degradation^58,68^. Research has mainly focused on its role in the pathogenesis of cancer or other neurodegenerative disorders, such as Parkinson’s disease^69,70^. Other proteins engaged in the Wnt signaling pathway, such as *BTRC* and *DIXDC1*, have been reported as ASD candidate genes^71–73^; however, *RNF146* was suggested as an ASD candidate gene only recently^74^. Our proteomic analyses of VPA-exposed mice and behavioral data from Rnf146-overexpressing mice suggest that Rnf146 is a strong candidate molecule responsible for social deficits associated with ASD.

The Wnt signaling pathway is critically involved in early vertebrate development, such as embryonic patterning and synaptic formation^75–77^. Thus, dysregulation of the Wnt/β-catenin signaling pathway is associated with many neurodevelopmental disorders, including autism^22,24,75^. Consistent with our finding that Wnt/β-catenin signaling is activated in VPA-exposed mice, upregulation of the Wnt signaling pathway has been reported to cause ASD-like behavioral phenotypes in rodents. For example, heterozygous GSK3β-knockout mice showed altered sociability and anxiety levels, paralleling the changes associated with chronic lithium administration, which activates Wnt/β-catenin signaling^78,79^. *In utero* injection of XAV939, a tankyrase inhibitor that subsequently activates β-catenin signaling, induced autism-like behaviors such as impaired sociability in mice^80^. A recent study reported that exposing a human organoid to VPA led to decreased expression of *Lhx9*, which has been suggested to negatively regulate Wnt signaling^81,82^. However, downregulation of the Wnt/β-catenin signaling pathway was also proposed as a candidate etiology of ASD in mouse models such as *Fmr1*^-/y^ mice^83,84^. These results suggest that bidirectional dysregulation of Wnt signaling is responsible for the etiology of ASD.

In addition to its role in development, Wnt signaling is also implicated in the adult brain in functions ranging from adult neurogenesis to synaptic plasticity^85,86^. Our finding that overexpressing Rnf146 in the adult PFC is sufficient to induce deficits in social behavior in mice strongly demonstrates the critical role of Wnt/β-catenin signaling in the adult PFC in regulating synaptic functions and social behaviors. We found that PFC neurons overexpressing Rnf146 displayed increased excitatory synaptic transmission, which is consistent with previous findings in VPA-exposed mice^38^. This increase in the amplitude and frequency of EPSCs without changes in IPSCs may lead to an E/I imbalance in the PFC of Rnf146-overexpressing mice, which in turn causes social deficits. Changes in excitatory synaptic transmission are highly correlated with the activated Wnt/β-catenin signaling pathway since this pathway contributes to the formation and functional regulation of central synapses^87^. Consistently, the AMPA-mediated synaptic current was shown to be regulated by postsynaptic β-catenin^88^.

Furthermore, our transcriptome analysis of the Rnf146-overexpressing PFC suggests that the expression of genes involved in synaptic organization and memory is significantly changed, which may underlie the observed increased excitatory transmission and social deficits in these mice. Interestingly, the expression of a component of the bone morphogenic protein (BMP) signaling pathway, *Chrd*, for which null mice showed an increased frequency of excitatory synaptic transmission, was significantly decreased in the Rnf146-overexpressing PFC (Supplementary Table 2a)^89^. Moreover, the expression of *Ube3b*, which is associated with intellectual disability^64,65^, was significantly decreased by Rnf146 overexpression (Supplementary Table 2a).

In this study, we used only male mice for proteomics and Rnf146 overexpression experiments because previous studies have shown that VPA exposure does not affect social behaviors in females^90,91^. In addition, Rnf146 was listed as one of the male-specific ASD candidate genes in a previous study^74^. Nevertheless, it would be intriguing to test whether Rnf146 overexpression in the PFC affects social behaviors in female mice in future studies.

Overall, our results provide novel insight into the molecular mechanism of prenatal drug exposure-associated ASD and suggest that the Rnf146-Wnt-β-catenin signaling pathway is a promising target to alleviate social deficits in ASD, even in adults.

## Supplementary information

Supplementary information

Supplementary Table 1

Supplementary Table 2

## Data Availability

All the data necessary to reach the conclusions of this paper are presented in the paper and/or the Supplementary Materials. The raw proteome MS data and MaxQuant search results have been deposited in the PRIDE database (project accession: PXD036413). Sequencing data have been deposited in GEO under accession code GSE215871.
