# Supplementary material for: Dysregulation of the Wnt/β-catenin signaling pathway via Rnf146 upregulation in a VPA-induced mouse model of autism spectrum disorder
Source: Exp Mol Med. 2023 Aug 1;55(8):1783–94. doi: 10.1038/s12276-023-01065-2 (PMC10474298; doi:10.1038/s12276-023-01065-2)
Supplement: Supplementary file 1 — Supplementary information [file 12276_2023_1065_MOESM1_ESM.pdf]

**Supplementary Information**

**This file includes:**

Supplementary Figs. 1 – 7

Supplementary Table 3

Supplementary methods

References

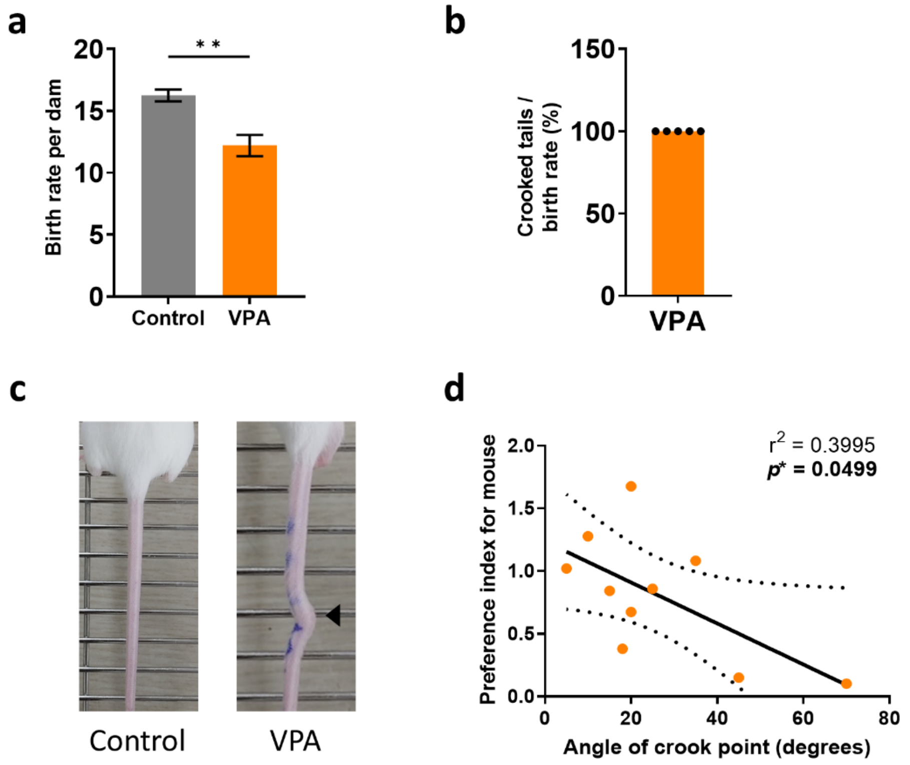


**Supplementary Fig. 1: Characterization of *in utero* VPA-exposed mice**

**a** VPA-exposed dam exhibited significantly lower birth rate than control dam (n = 4 for vehicle dam, n = 5 for VPA- exposed dam, two-tailed unpaired t test; $t_7$ = 3.813, $**P$ = 0.0066). **b** VPA-exposed mice are highly likely show the crooked tail phenotype. **c** Representative images of control and VPA-exposed mice tail. Compared to the control mouse, VPA-exposed mouse showed a clear tail malformation. **d** Correlation between mice social preference index and angles of crooked point as degrees. Pearson correlation coefficients was used to evaluate each linear correlation. Each linear regression line is shown with 95% confidence bands (two dotted lines). The degree of significant correlation ($p$) and goodness of fit ($r^2$) were described in each figure.

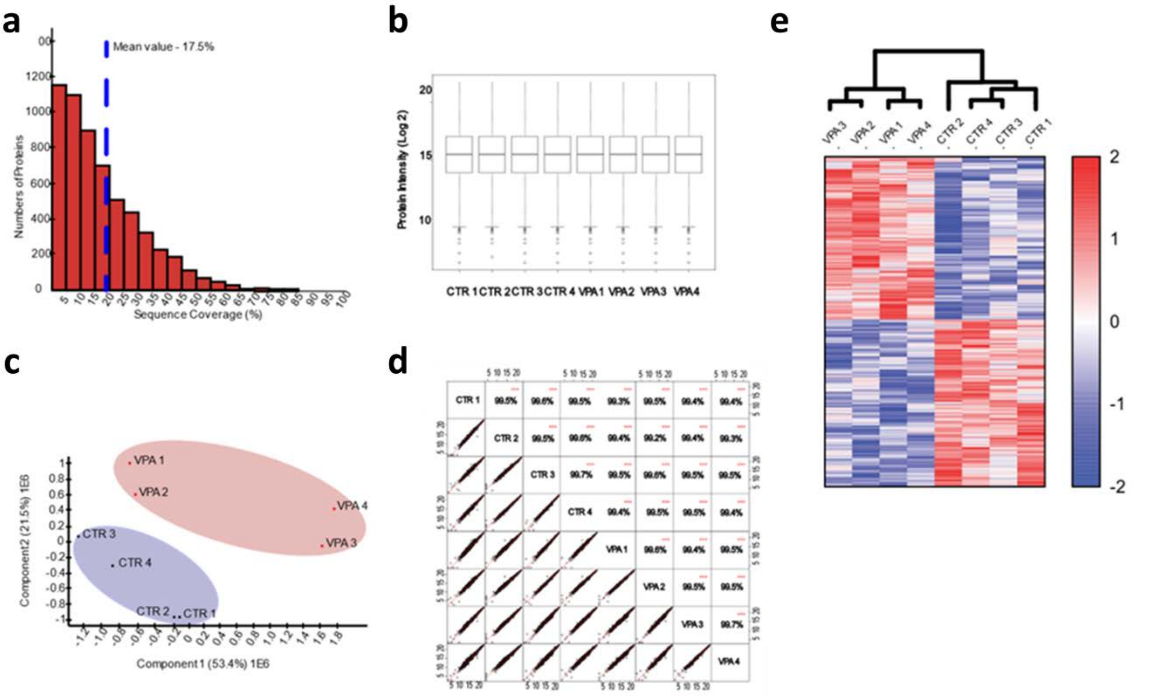

**Supplementary Fig. 2: Quantitative proteomics result of mPFC of VPA-exposed mice**

**a** The histogram showed ~17.5% average sequence coverage of the proteome identified. Quantile normalization (**b**) showed a good reproducible correlation between biological replicates. **c** Principal component analysis resulted in a nearly complete separation of proteome expression between VPA-exposed and control mice. **d** Pearson correlation coefficients between these samples demonstrate good reproducibility. **e** The hierarchical clustering was carried out using DEPs.

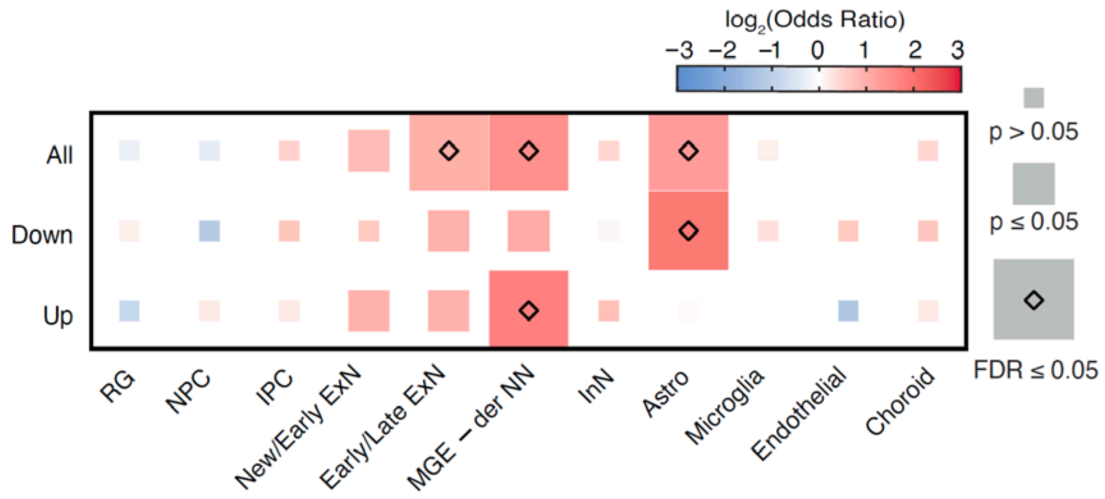


**Supplementary Fig. 3: Enrichment analysis of VPA-DEPs with genes specific to human cortex cell types.**

Enrichment analysis of VPA-DEPs with genes specific to 11 cell types defined by single-cell RNA-seq data obtained from the developing human cortex samples. The $log_2$(odds ratio) values that exceeded the range of -3 to 3 were fitted within this the range. The blue and red colors represent the $log_2$(odds ratio) values, and the size of the box corresponds to the significance in enrichment. Radial glia (RG), Neural progenitor cell (NPC), Intermediate progenitor cell (IPC), Excitatory neuron (ExN), Medial ganglionic eminence (MGE), Newborn neuron (NN), Inhibitory neuron (InN)

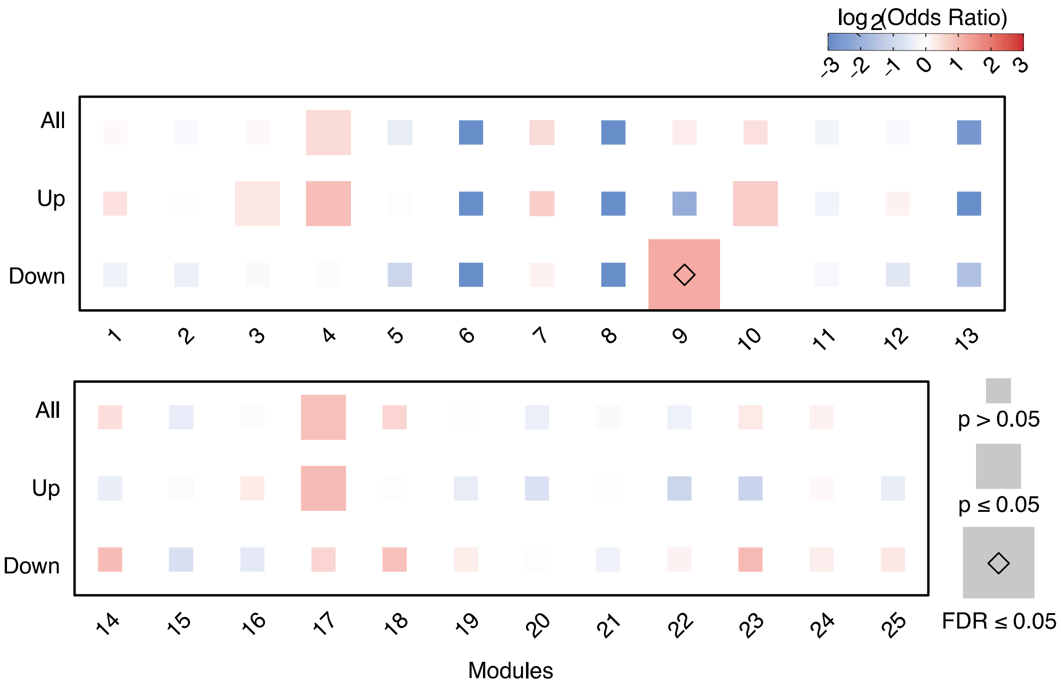


**Supplementary Fig. 4: Enrichment analysis of VPA-DEPs with coexpressed module genes from postmortem cortex of ASD humans.**

Enrichment analysis of VPA-DEPs with 25 coexpressed module genes identified by WGCNA from postmortem cortex of humans diagnosed as ASD. The $\log_2$(odds ratio) values that exceeded the range of -3 to 3 were fitted within this the range. The blue and red colors represent the $\log_2$(odds ratio) values, and the size of the box corresponds to the significance in enrichment.

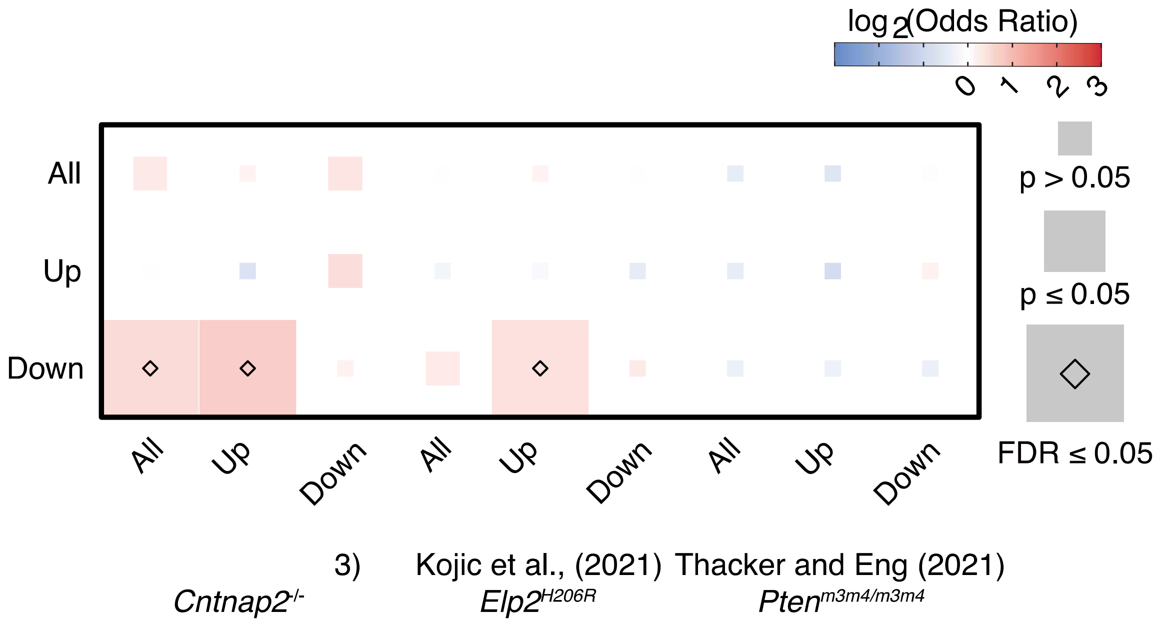


**Supplementary Fig. 5: Enrichment analysis of VPA-DEPs with identified DEPs in previous ASD proteomics studies.**

Enrichment analysis of VPA-DEPs with identified DEPs in three previously reported ASD proteomics studies. The $\log_2$(odds ratio) values that exceeded the range of -3 to 3 were fitted within this the range. The blue and red colors represent the $\log_2$(odds ratio) values, and the size of the box corresponds to the significance in enrichment.

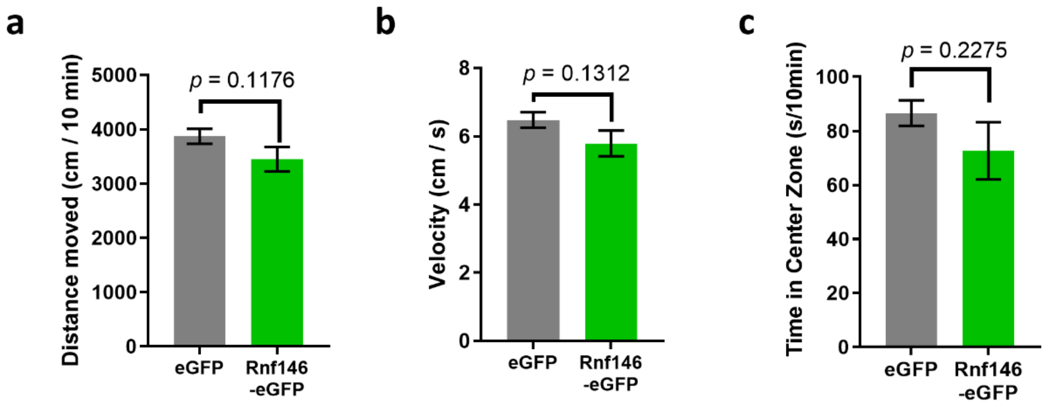


**Supplementary Fig. 6: Rnf146 overexpression did not affect mice basal locomotor activity**

**a** Rnf146-overexpressing mice showed comparable moving distance to eGFP-overexpressing mice (n = 9 for eGFP, n = 8 for Rnf146, two-tailed unpaired t test; $t_{15}$ = 1.66, $P$ = 0.1176).

**b** Rnf146-overexpressing mice showed comparable speed to eGFP-overexpressing mice (n = 9 for eGFP, n = 8 for Rnf146, two-tailed unpaired t test; $t_{15}$ = 1.597, $P$ = 0.1312).

**c** Rnf146-overexpressing mice showed basal anxiety level comparable to eGFP-expressing mice (n = 9 for eGFP, n = 8 for Rnf146, two-tailed unpaired t test; $t_{15}$ = 1.258, $P$ = 0.2275).

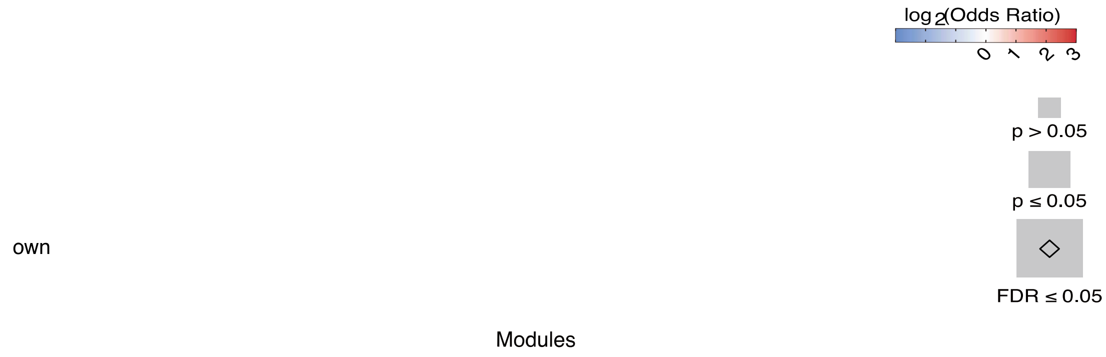


**Supplementary Fig. 7: Enrichment analysis of Rnf146-DEGs with coexpressed module genes (Module 1-14).**

Enrichment analysis of Rnf146-DEGs with 51 coexpressed module genes identified by WGCNA. Enrichment results for WGCNA modules 1-14 are representatively depicted. The $\log_2$(odds ratio) values that exceeded the range of -3 to 3 were fitted within this the range. The blue and red colors represent the $\log_2$(odds ratio) values, and the size of the box corresponds to the significance in enrichment.

**Supplementary Table 3: Electrophysiological effect of Rnf146 overexpression in prefrontal pyramidal neurons**

| Properties | eGFP | | Rnf146 | | Statistics | | | |
|---|---|---|---|---|---|---|---|---|
| | Mean | S.E.M | Mean | S.E.M | Method | P value | Significantly different (P<0.05)? | t, df |
| Holding current (pA) | 4.264 | 9.381 | -6.741 | 10.14 | Unpaired t test | 0.4414 | No | t=0.7813 df=27 |
| Rheobase (pA) | 92.31 | 17.76 | 96.88 | 9.649 | Unpaired t-test | 0.814 | No | t=0.2376 df=27 |
| Cm (pF) | 92.08 | 17.51 | 110.7 | 11.15 | Unpaired t-test | 0.3614 | No | t=0.9285 df=27 |
| RMP (mV) | -65.09 | 2.389 | -64.69 | 2.245 | Unpaired t-test | 0.9031 | No | t=0.1229 df=27 |

## Supplementary Methods

### Viral vectors and AAV packaging

Adeno-associated viral (AAV) vector for neuronal expression of Rnf146 (pAAV-hsyn-mRnf146-T2A-eGFP-WPRE) was constructed by recombinant PCR as follows: mouse Rnf146 (mRnf146) cDNA was amplified from CMV-Tag5-mRNF146-myc[1] by PCR using the following primers: 5’ - GTA **GCT AGC** CAG GAA TTC ATG GCC GG- 3’ and 5’-CTC CAC GTC ACC GCA TGT TAG AAG ACT TCC TCT GCC CTC AAC CTC TG T CAC TGT GCA-3’. T2A-eGFP was amplified from AAV-hsyn-T2A-eGFP using the following primers: 5’-CTA ACA TGC GGT GAC GTG GA-3’ and 5’-CCC **AAG CTT** ACT TGT ACA GCT CGT CC-3’. T2A sequence are underlined, and the restriction enzyme sites are in bold. The PCR product was digested with Nhe I and Hind III (Enzynomics, South Korea) and ligated into the pAAV-hsyn-tdtomato-WPRE. CMV-Tag5-mRNF146-myc was a kind gift from Prof. Joo-Ho Shin (SKKU, South Korea). AAV was prepared as previously described [2]. Briefly, pAAV-hsyn-mRNF146-T2A-eGFP-WPRE or pAAV-hsyn-eGFP-WPRE (13ug each) was co-transfected with p5E18-RxC1 (13ug) and pAd-ΔF6 plasmid (26 μg) into HEK293T cells using polyethylenimine (PEI, Polysciences, Inc., USA). After 72 hours, culture media were collected and AAVs were purified by ultracentrifugation (Beckman Coulter, USA). The harvested solution diluted in DPBS (LB001-02, Welgene, South Korea) was filtered with the Amicon ultra-15 filter tube (UFC910024, Millipore, USA) and washed 3 times with DPBS. The titer of viral products was quantified by quantitative PCR.

### LC-MS/MS analysis

The prefrontal cortex tissues were lysed in a lysis buffer containing 9 M urea in 20 mM HEPES (pH 7.5) with phosphatase inhibitor (PhosSTOP, Roche, Penzberg, Germany, 4906845001) in HPLC grade water by applying probe sonication on ice for 5 min. A total of 200 µg proteins from each sample was used for analysis. The disulfide bonds were reduced by 10 mM of 1,4-dithiothreitol (DTT) for 30 min and alkylated using 30 mM iodoacetamide (Sigma-Aldrich, St. Louis, MO, USA, I1149) in the dark for 30 min. Samples were diluted with 10 mM of triethylammonium bicarbonate (Sigma-Aldrich, St. Louis, Mo, USA, T7408) to lower the urea concentration to 1 M. Proteins were digested with MS-grade trypsin (Thermo Fisher Scientific, 90058) with a protein to enzyme ratio of 50 to 1 for 12 hours at 37°C. Enzymatic reaction was quenched with trifluoroacetic acid (Thermo Fisher Scientific, 28904). After desalting, peptides were chemically labelled using a 10-plex tandem mass tag in 4 VPA induced mice (127N,127C,128N, and 128C) and 4 control mice (129N, 129C, 130N, and 130C) (TMT, Thermo Fisher Scientific, 90110). The reaction proceeded for 2 hours at room temperature with an intermediate vortex and spun down every 15 mins. The TMT-labeled peptides were dried and resuspended with 10 mM ammonium bicarbonate (Sigma-Aldrich, St. Louis, MO, USA, 285099) and then pooled for a basic pH reverse-phase liquid chromatography fractionation using the 1260 Infinity II LC system (Agilent). Pooled peptides were loaded onto a separation column (C18, 4 µm pore size, 4.6 mm × 250 mm, Accucore XL, Thermo Fisher Scientific), fractionated into a 96-well plate, and then non-contiguously concatenated into 24 fractions. Fractionated peptides were dried using a speed-vac and stored at -80°C for LC-MS/MS analysis.

Each fraction was analysed on an LC-MS/MS consisting of an EASY-nLC (Thermo Fisher Scientific) nanoflow liquid chromatography system and an Orbitrap Q-Exactive mass spectrometry (Thermo Fisher Scientific) with an EASY-Spray ion source. The peptides were resuspended in HPLC grade water with 0.1% formic acid (FA), and 2 µg were loaded onto a trap column (100 µm inner diameter, 2 cm, 5 µm C18 particles, Acclaim PepMap100, Thermo

Fisher Scientific) and then separated on an EASY-Spray HPLC column (75 µm inner diameter, 50 cm, and 2 µm C18 particles, PepMap RSLC, Thermo Fisher Scientific) at a flow rate of 250 nL/min. The mobile phase buffer contains 0.1% FA in HPLC grade water (Phase A) and 0.1% FA in 95% HPLC grade acetonitrile (phase B), with gradient elution of 5% B at 0-5 min; 5-25% at 5-85 min; 25-90% at 85-95 min; 90% at 95-105 min; 90-5% at 105-110 min; 5% at 110-120 min. MS analysis was performed in a data-dependent acquisition mode. The electrospray ionization voltage was at 2,400 V, while the capillary temperature was at 270 $^{o}$C. MS1, precursor ions and MS2, peptide fragmentation ions were acquired. The full scan MS1 was collected from 350 *m/z* to 2000 *m/z* with a resolution of 35,000, for dd-MS in MS2 mode first ms value was set to 100 *m/z*, and was collected from 200 *m/z* to 2000 *m/z* with a resolution of 70,000 for dd-MS2. The isolation window was set to 1.4 *m/z.* The maximum injection time for MS1 at 100 ms and MS2 at 50 ms. The normalized collision energy was set to 29.

The data obtained from mass spectrometry was analyzed with the MaxQuant software (version 1.6.1.0) with UniProt/SwissProt mus musculus sequence database (released on 10/18/2020 containing 21909 canonical sequences)[3]. In group-specific parameters, reporter ion MS2 was selected and filtered. 10 plex TMT isobaric labels were selected afterwards. The reporter mass tolerance was set to 0.003 and a filter by PIF was selected. The carbamidomethyl on cysteine was set for fixed modification and in variable modifications oxidation and acetyl were selected. The false discovery rates at the level of protein and peptide-spectrum matches (PSM) were set to 0.01. The contamination and reverse-identified proteins were removed from the results obtained by MaxQuant. TMT-based expression data were quantile-normalized as described previously[4]. The principal component analysis, multi-scatter plot analysis, and hierarchical clustering of proteins were performed.

**Enrichment Analyses of VPA-DEPs**

We performed a hypergeometric enrichment test with cell types using a single-cell dataset generated from a mouse nervous system[5]. To define cell type-enriched genes, we selected 100 top marker genes based on pre-computed enrichment scores from the original study. Further hierarchical clustering of provided cell types was manually conducted, resulting in five cell type categories: Excitatory neuron (TEGLU1-24), Inhibitory neuron (TEINH1-21), Oligodendrocyte (OPC, NFOL1-2, COP1-2, MOL1-3, MFOL1-2), Astrocyte (ACTE1-2), and Microglia (MGL1-3). All marker genes of the cell type included in each category were subject to a cell type enrichment test.

We further performed a hypergeometric enrichment test with cell types using a single cell dataset generated from a developing human cortex sample[6]. To define cell type-enriched genes, we calculated a tau metric[7] from the per gene log2TPM of log2UMI values for genes within cell type clusters as instructed in our previous study[8]. Associated cell types were combined based on the results of hierarchical clustering in a recent study[9]. Across cell-type-specific clusters, each gene was ranked by the TPM to UMI ratio, and the top 300 genes selected for each cell type were subject to the cell type enrichment test.

To test the enrichment with neurodevelopment and neurological disorders, we collated the list of risk genes from previous genetic association studies of the disorders. Autism spectrum disorder (ASD) risk genes were collected from Fu et al.[10], which refined high-confidence genes (n = 185) with exome sequencing-based gene discovery at a false discovery rate (FDR) $\leq$ 0.05. From Heyne et al.[11], we selected high-confidence epilepsy candidate genes (n = 33), where multiple de novo variants were seen. Genes associated with developmental delay (n = 285) were obtained from the latest exome sequencing study of the Deciphering Developmental Disorders project[12]. We obtained schizophrenia (SCZ) risk genes (n = 32) from Singh et al.[13] implicated at a false discovery rate (FDR) $\leq$ 0.05 with whole exomes meta-analysis.

We tested the enrichment of VPA-DEPs with differentially expressed genes (DEGs) and co-expressed module genes from the postmortem cortex of ASD patients using a hypergeometric enrichment test[14]. Only protein-coding genes were selected from the given data, and all genes at a FDR ≤ 0.05 were considered ASD DEGs (n = 1057). DEPs identified in other ASD proteomic analyses were obtained from three distinct studies[15-17]. The evaluation of DEPs was conducted based on the thresholds established in each corresponding study. In addition, we chose to use a one-sided test instead of a two-sided test as we were not interested in cases where the enrichment was significantly lower than expected in the Fisher test.

**Functional mapping and Protein-protein interaction (PPI) network**

We evaluated the biological functions of the VPA-DEP homologs onto the pathway terms from the Reactome Database[18] using the EnrichR tool[19]. To construct a protein-protein interaction (PPI) network of VPA-DEPs associated with the Wnt signaling and neurodevelopmental pathways, we utilized interaction pairs from the STRING database (v11.0)[20] and selected high-quality pairs defined by combined scores greater than 700. The network was visualized by the Cytoscape software[21].

**Enrichment Analyses of DEGs in RNF146 overexpression mice**

Raw reads were processed with Cutadapt[22] for Illumina adapter trimming and removal of low-quality reads. We removed 15 bp of each read for low quality (q < 30) and discarded the reads shorter than 100 base pairs. After the quality check with FastQC (http://www.bioinformatics.babraham.ac.uk/projects/fastqc/), we quantified the abundance of transcripts using Salmon software (v1.9.0)[23] in quasi-mapping-based mode onto the mouse reference transcriptome (GENCODE vM30, GRCm39). Quantified transcript-level estimates

were imported to R using tximport and were converted to gene-level counts based on gene IDs from GENCODE version 30 mouse genome annotation file[24].

After filtering low-level counts, we performed differential gene expression analysis with DESeq2 (v1.36.0)[25]. Read counts were automatically normalized using the `DESeq` function by dividing with size factors and fitting to a negative binomial distribution. We performed variance stabilizing transformation of the count data using the function `vst` from DESeq2 and removed variations associated with batch effects using the `removeBatchEffect` function from limma R package (v3.48.3)[26]. Genes with an FDR under 0.1 were considered as differentially expressed genes (Rnf146-DEGs).

Pre-ranked enrichment test of the Rnf146-DEGs onto pathway terms obtained from MsigDB Gene Ontology was performed using R package FGSEA. The activities of 14 pathways were estimated with PROGENy R package (v1.18.0)[27] based on the expression signatures of 100 responsive genes that were most consistently perturbed by the activation of pathways in the previous experiments. The scores of each pathway were scaled to have a mean of zero and a standard deviation of one. Enrichment test with synaptic locations of the brain was performed using the SynGO web-based tool[28]. Pathway terms were obtained from MsigDB Gene Ontology Cell Components. The activities of transcription factors were estimated with DoRothEA R package (v1.6.0) based on the expression profiles of curated target genes for each transcription factor[29]. Enrichment for transcription factor activities was evaluated using the statistics from the differential expression analysis results.

**WGCNA network construction and module identification**

To identify the functional topology in samples, Weighted Gene Co-Expression Network Analysis (WGCNA, version 1.71) was applied to transcriptomics data. From 54,778 genes, genes with missing entries, entries with weight below a threshold (minRelativeWeight

= 0.1), genes with zero-variance were removed, and only 22,715 genes with a mean expression value of 1 or more were used in WGCNA. We performed network construction, using a signed network, choosing the soft-thresholding power, the scale-free topology 0.8 or higher and the mean connectivity less than 100. To calculate the topology overlap similarity matrix from expression data, we applied the 'TOMsimilarityFromExpr' function, which uses the Pearson's correlation method. To generate the network dendrogram, average linkage hierarchical clustering of the topological overlap dissimilarity matrix (1-TOM) was used. Modules were identified using the cutreeHybrid function, which detect modules in a dendrogram produced by the hclust function, with the following parameters: minimum module size =200, height cut = 0.999, negative pamStage, and deepSplit =4 (the highest sensitivity). Additionally, to merge modules that are too close to distinguish, the mergeCloseModules function were used, and the criterion for merging modules is when the maximum dissimilarity (i.e., 1-correlation) is lower 0.15 (cutHeight = 0.15). We characterized WGCNA module genes using the gProfiler R package, describing the module-specific biological term with the size of term between 100 and 500, and performed a hypergeometric enrichment test of WGCNA module genes with Rnf146-DEGs. Hub genes of module 1 and module 2 were ranked by the intramodular connectivity, and the network of hub genes were visualized using the R package igraph (https://igraph.org).

**Social Behavior Test**

Eight weeks old male VPA- or saline-exposed mice were subjected to the 3-chamber social behavior test as previously described[30]. An acrylic box (Length 1001mm × Width 416mm × Height 220mm) without a lid is divided into three compartments with two acrylic walls, allowing a mouse to freely explore between compartments. Same shape but slightly different sized acrylic box (Length 600mm × Width 400mm × Height 200mm) was used for prefrontal

RNF146-overexpressed mice. For social preference test, a subject mouse was placed into the chamber of which one side is occupied by 6 weeks old conspecific social target and the other side is empty or occupied by mouse-shaped toy object for 10 minutes. After 10 minutes, the subject mouse was led in the middle chamber while the empty cup is replaced by a cup with a novel conspecific for social recognition test for another 10 minutes. Subject's behavior was recorded during the social behavior test and analyzed afterward. Experimenters were blind to the experimental conditions throughout the experiments. Mice behavior within the habituation session was monitored using an automated mouse-tracking software (Ethovision XT 11.5, Noldus, Netherlands). Behavior data from animals which showed a biased preference (>75%) towards one empty chamber than the other chamber during habituation session were discarded. Data from one mouse which showed an abnormal hypoactivity during the habituation session was also excluded from analysis. Social behaviors of VPA-exposed and control mice were analyzed automatically by measuring the cumulative duration within 5 cm circular zone around the target cup using Ethovision software. Social behaviors of RNF146-overexpressed and eGFP-overexpressed mice were manually scored. Preference index (PI) was calculated by following equations ($ET_M$: exploration time for a mouse; $ET_O$: exploration time for an object; $ET_N$: exploration time for a novel mouse; $ET_F$: exploration time for a familiar mouse).

$$\text{PI for social preference} = \frac{\mathrm{ET_M} - \mathrm{ET_0}}{\mathrm{ET_M} + \mathrm{ET_0}}$$

$$\text{PI for social recognition} = \frac{\mathrm{ET_N} - \mathrm{ET_F}}{\mathrm{ET_N} + \mathrm{ET_F}}$$

**Open field test**

Subject mice were placed in a white acrylic box (Length 330 mm × Width 330 mm × Height 330 mm) to move freely for 10 minutes. The center zone was set in size of length 200 mm ×

width 200 mm size. The movements of subject mice were tracked automatically by EthoVision XT 11.5 (Noldus).

**Electrophysiology**

Electrophysiological recordings were performed as described previously [31]. Coronal slices with prefrontal cortex as 300 μm thickness were obtained by a vibratome (VT1200s, Leica)) after isoflurane anesthesia followed by decapitation. Brain slices were cut in ice-cold cutting solution containing (in mM): 93 N-Methyl-D-glucamine (NMDG), 2.5 KCl, 10 MgSO4, 0.5 CaCl2, 1.25 NaH2PO4, 30 NaHCO3, 25 glucose, 10 HEPES, 5 Na ascorbate, 2 thiourea, 3 Na pyruvate, 12 L- acetyl - cysteine, perfused with 95% O2 and 5% CO2. Slices were immediately transferred to the same cutting solution at 32°C and incubated for 10 minutes before transferred to room temperature artificial cerebrospinal fluid (ACSF) containing (in mM): 125 NaCl, 2.5 KCl, 1 MgCl2, 2 CaCl2, 1.25 NaH2PO4, 26 NaHCO3, 10 glucose, perfused with 95% O2 and 5% CO2. Brain slices were recovered in ACSF for 1 hour before the recording and placed in a submerged recording chamber perfused with ACSF for at least 10 minutes before recording. All recordings were made at 32°C. $K^+$ gluconate internal solution containing (in mM): 135 K-gluconate, 5 KCl, 2 NaCl, 10 HEPES, 0.1 EGTA, 5 Mg ATP, 0.4 Na3 GTP and 10 Tris(di) phosphocreatine (pH 7.20 adjusted by KOH) was filled in recording glass pipettes (4-5MΩ). Data were acquired using an EPC-10 patch-clamp amplifier (HEKA Elektronik) and PatchMaster software (HEKA Elektronik) with a 20kHz sampling rate while the signals were filtered at 2 kHz. Excitability and synaptic current data were analyzed by customized LabView (National Instruments) analysis programs[32].

## Reference


1 Andrabi, S. A. *et al.* Iduna protects the brain from glutamate excitotoxicity and stroke by interfering with poly(ADP-ribose) polymer-induced cell death. *Nat. Med.* **17**, 692-699 (2011).

2 Ryu, H.-H. *et al.* Excitatory neuron–specific SHP2-ERK signaling network regulates synaptic plasticity and memory. *Science Signaling* **12**, eaau5755 (2019).

3 Cox, J. & Mann, M. MaxQuant enables high peptide identification rates, individualized p.p.b.-range mass accuracies and proteome-wide protein quantification. *Nature Biotechnology* **26**, 1367-1372 (2008).

4 Franks, J. M., Cai, G. & Whitfield, M. L. Feature specific quantile normalization enables cross-platform classification of molecular subtypes using gene expression data. *Bioinformatics* **34**, 1868-1874 (2018).

5 Zeisel, A. *et al.* Molecular Architecture of the Mouse Nervous System. *Cell* **174**, 999-1014 e1022 (2018).

6 Nowakowski, T. J. *et al.* Spatiotemporal gene expression trajectories reveal developmental hierarchies of the human cortex. *Science* **358**, 1318-1323 (2017).

7 Kryuchkova-Mostacci, N. & Robinson-Rechavi, M. A benchmark of gene expression tissue-specificity metrics. *Brief Bioinform.* **18**, 205-214 (2017).

8 Werling, D. M. *et al.* Whole-Genome and RNA Sequencing Reveal Variation and Transcriptomic Coordination in the Developing Human Prefrontal Cortex. *Cell. Rep.* **31**, 107489 (2020).

9 Satterstrom, F. K. *et al.* Large-Scale Exome Sequencing Study Implicates Both Developmental and Functional Changes in the Neurobiology of Autism. *Cell* **180**, 568-584 e523 (2020).

10 Fu, J. M. *et al.* Rare coding variation provides insight into the genetic architecture and phenotypic context of autism. *Nat. Genet.* **54**, 1320-1331 (2022).

11 Heyne, H. O. *et al.* De novo variants in neurodevelopmental disorders with epilepsy. *Nat. Genet.* **50**, 1048-1053 (2018).

12 Kaplanis, J. *et al.* Evidence for 28 genetic disorders discovered by combining healthcare and research data. *Nature* **586**, 757-762 (2020).

13 Singh, T. *et al.* Rare coding variants in ten genes confer substantial risk for schizophrenia. *Nature* **604**, 509-516 (2022).

14 Parikshak, N. N. *et al.* Genome-wide changes in lncRNA, splicing, and regional gene expression patterns in autism. *Nature* **540**, 423-427 (2016).

15 Jang, W. E. *et al.* Cntnap2-dependent molecular networks in autism spectrum disorder revealed through an integrative multi-omics analysis. *Molecular Psychiatry* **28**, 810-821 (2023).

16 Kojic, M. *et al.* Elp2 mutations perturb the epitranscriptome and lead to a complex neurodevelopmental phenotype. *Nature Communications* **12**, 2678 (2021).

17 Thacker, S. & Eng, C. Transcriptome-(phospho)proteome characterization of brain of a germline model of cytoplasmic-predominant Pten expression with autism-like phenotypes. *NPJ Genom. Med.* **6**, 42 (2021).

18 Jassal, B. *et al.* The reactome pathway knowledgebase. *Nucleic Acids Res* **48**, D498-D503 (2020).

19 Kuleshov, M. V. *et al.* Enrichr: a comprehensive gene set enrichment analysis web server 2016 update. *Nucleic Acids Research* **44**, W90-W97 (2016).

20 Szklarczyk, D. *et al.* STRING v11: protein-protein association networks with increased coverage, supporting functional discovery in genome-wide experimental datasets.

*Nucleic Acids Res.* **47**, D607-D613 (2019).
21 Shannon, P. *et al.* Cytoscape: a software environment for integrated models of biomolecular interaction networks. *Genome Res.* **13**, 2498-2504 (2003).
22 Kechin, A., Boyarskikh, U., Kel, A. & Filipenko, M. cutPrimers: A New Tool for Accurate Cutting of Primers from Reads of Targeted Next Generation Sequencing. *J Comput. Biol.* **24**, 1138-1143 (2017).
23 Patro, R., Duggal, G., Love, M. I., Irizarry, R. A. & Kingsford, C. Salmon provides fast and bias-aware quantification of transcript expression. *Nature Methods* **14**, 417-419 (2017).
24 Frankish, A. *et al.* GENCODE reference annotation for the human and mouse genomes. *Nucleic Acids Res.* **47**, D766-d773 (2019).
25 Love, M. I., Huber, W. & Anders, S. Moderated estimation of fold change and dispersion for RNA-seq data with DESeq2. *Genome Biology* **15**, 550 (2014).
26 Ritchie, M. E. *et al.* limma powers differential expression analyses for RNA-sequencing and microarray studies. *Nucleic Acids Research* **43**, e47-e47 (2015).
27 Schubert, M. *et al.* Perturbation-response genes reveal signaling footprints in cancer gene expression. *Nature Communications* **9**, 20 (2018).
28 Koopmans, F. *et al.* SynGO: An Evidence-Based, Expert-Curated Knowledge Base for the Synapse. *Neuron* **103**, 217-234.e214 (2019).
29 Garcia-Alonso, L., Holland, C. H., Ibrahim, M. M., Turei, D. & Saez-Rodriguez, J. Corrigendum: Benchmark and integration of resources for the estimation of human transcription factor activities. *Genome Res.* **31**, 745 (2021).
30 Kim, J.-W. *et al.* Pharmacological modulation of AMPA receptor rescues social impairments in animal models of autism. *Neuropsychopharmacology* **44**, 314-323 (2019).
31 Park, G. *et al.* Social isolation impairs the prefrontal-nucleus accumbens circuit subserving social recognition in mice. *Cell Reports* **35** (2021).
32 Kim, Y. G., Shin, J. J. & Kim, S. J. Minhee Analysis Package: an integrated software package for detection and management of spontaneous synaptic events. *Molecular Brain* **14**, 138 (2021).
